# Perspective: The viscoelastic properties of biofilm infections and mechanical interactions with phagocytic immune cells

**DOI:** 10.3389/fcimb.2023.1102199

**Published:** 2023-02-16

**Authors:** Marilyn Wells, Rebecca Schneider, Bikash Bhattarai, Hailey Currie, Bella Chavez, Gordon Christopher, Kendra Rumbaugh, Vernita Gordon

**Affiliations:** ^1^Department of Physics, Center for Nonlinear Dynamics, The University of Texas at Austin, Austin, TX, United States; ^2^Department of Surgery, Texas Tech University Health Sciences Center, Lubbock, TX, United States; ^3^Department of Mechanical Engineering, Texas Tech University, Lubbock, TX, United States; ^4^LaMontagne Center for Infectious Disease, The University of Texas at Austin, Austin, TX, United States; ^5^Interdisciplinary Life Sciences Graduate Program, The University of Texas at Austin, Austin, TX, United States

**Keywords:** biofilm, viscoelasticity, rheology, phagocytosis, neutrophil, immune system

## Abstract

Biofilms are viscoelastic materials that are a prominent public health problem and a cause of most chronic bacterial infections, in large part due to their resistance to clearance by the immune system. Viscoelastic materials combine both solid-like and fluid-like mechanics, and the viscoelastic properties of biofilms are an emergent property of the intercellular cohesion characterizing the biofilm state (planktonic bacteria do not have an equivalent property). However, how the mechanical properties of biofilms are related to the recalcitrant disease that they cause, specifically to their resistance to phagocytic clearance by the immune system, remains almost entirely unstudied. We believe this is an important gap that is ripe for a large range of investigations. Here we present an overview of what is known about biofilm infections and their interactions with the immune system, biofilm mechanics and their potential relationship with phagocytosis, and we give an illustrative example of one important biofilm-pathogen (*Pseudomonas aeruginosa*) which is the most-studied in this context. We hope to inspire investment and growth in this relatively-untapped field of research, which has the potential to reveal mechanical properties of biofilms as targets for therapeutics meant to enhance the efficacy of the immune system.

## Introduction

A biofilm is a community of microorganisms which produces a matrix of extracellular polymeric substances (EPS) composed of self-produced polymers, proteins, extracellular nucleic acids (both eDNA and eRNA), and various biomolecules that function as nutrients or virulence factors (Flemming et al., 2007; Maunders and Welch, 2017; Karygianni et al., 2020). This matrix provides microbes protection against external threats such as antibiotic treatment and immune clearance, leading to persistent and dangerous infections (Costerton et al., 1999). Biofilm-associated infections are commonly found in the lungs of people with cystic fibrosis, in chronic wound beds, and on surfaces foreign to the body such as implants, catheters, prosthetics, and contact lenses. Despite continued advancements in research and medicine, chronic bacterial infections continue to be a major concern in healthcare. Chronic infections have a substantial cost, in the millions of dollars for healthcare systems and in the millions of lives impacted (Versey et al., 2021; Eriksson et al., 2022). In addition to a heavy toll on quality of life, chronic infections further the socioeconomic divide, through increased mortality rates, extensive hospitalization, loss of employment, economic burdens, and patient suffering (Eriksson et al., 2022; Thomsen et al., 2022). Most chronic infections, up to 80%, are caused by biofilms (González et al., 2018; Moser et al., 2021).

Biofilms can vary greatly, both in structure and composition, due to environmental factors and the identity of their microbial constituents. The EPS itself is highly dependent on microbial species and environmental stresses, including shear force, nutrient scarcity, and in the case of infections, host response and antibiotic pressure (Karygianni et al., 2020). In addition to self-produced EPS, biofilms incorporate host material, including components of the wound bed such as collagen and fibrin (Donlan, 2002; Rahman et al., 2022a), and DNA released from dead immune cells responding to the infection (Walker et al., 2005). The EPS provides a physical barrier which limits antibiotic efficacy primarily by blocking access to the cells within the biofilm. Biofilms are viscoelastic materials, exhibiting both solid-like and fluid-like characteristics. These properties vary on the bulk level due to the presence of particular components of the EPS matrix (Kovach et al., 2020), and on the microscopic level as biofilms are structurally inhomogeneous (Donlan, 2002). Studies have shown that treatments designed to structurally compromise established biofilms can effectively alter the biofilm’s mechanical properties, including the elastic modulus G’, toughness, yield strain, and yield stress (Kovach et al., 2020).

While biofilm tolerance to antimicrobial treatments has been researched extensively (Stewart and Costerton, 2001; Stewart, 2002), here we discuss motivation for investigating the mechanical clearance of biofilms by host immune cells. Neutrophils are phagocytic immune cells which act as the body’s first line of defense against infection. Recruited chemotactically to the infection site, neutrophils employ three primary mechanisms for killing pathogens: phagocytosis, degranulation, and NETosis (Segal, 2005). While degranulation and NETosis involve the expulsion of antimicrobial substances, phagocytosis involves the engulfment of individual microbes and subsequent exposure to bactericidal mechanisms within a phagosome (Uribe-Querol and Rosales, 2020). Neutrophils easily engulf planktonic cells and small aggregates of bacteria. However, the diameters of aggregates characterizing biofilm infections can be as large as approximately 100 μm, which is an order of magnitude greater than the diameter of a neutrophil (Kirketerp-Moller et al., 2008; Rudkjobing et al., 2012; Kragh et al., 2014; Stewart, 2014). When faced with such a target, neutrophils often experience “frustrated” phagocytosis (Herant et al., 2006), leading to an inflammatory response that can ultimately be harmful to the host. We have recently shown, for abiotic gels that recreate the mechanical properties and lengthscales of biofilm infections, the phagocytic success of neutrophils is dependent on the mechanics of the target and on the timescale of phagocytic interactions (Davis-Fields et al., 2019; Bakhtiari et al., 2021).

Overall, biofilm infections are debilitating burdens for both patients and healthcare systems (Versey et al., 2021). Microbes utilize biofilms with a protective EPS matrix to survive in potentially hostile environments, including human hosts (Flemming et al., 2007; Maunders and Welch, 2017; Karygianni et al., 2020). Biofilms are difficult to treat for numerous reasons including strong physical adherence, high antibiotic tolerance, immune evasion, and immune response antagonism (Gunn et al., 2016; González et al., 2018; Moser et al., 2021; Versey et al., 2021; Thomsen et al., 2022). Chronic infections will continue to be a costly problem until new treatment methods are developed to counteract and combat biofilm formation. Here, we present our line of reasoning to support our argument that biofilm mechanics may have an important role in immune evasion and that knowledge of the relationship between the composition and mechanics of biofilms and the success of phagocytic clearance could reveal cases in which mechanical properties of biofilms could be targeted by therapeutic approaches intended to enhance the efficacy of the body’s own mechanisms for phagocytic clearance.

## Main body

### Background

#### Host immune response to bacterial infection

For individuals with a fully-functioning immune system who contract a planktonic bacterial infection, the infection is cleared by initial interactions with the nonselective innate immune system and if necessary, by further involvement from the highly selective adaptive immune response (González et al., 2018). However, in biofilm infections, this progressive immune response, incorporating both the innate and adaptive immune systems, is used against the host to stimulate constant and ineffective inflammation, damaging surrounding tissue and worsening the infection (Moser et al., 2021; Thomsen et al., 2022). This effect can clearly be seen in chronic wound biofilm infections, which are inundated by perpetual inflammation (McCallon et al., 2014; Eriksson et al., 2022). Recovering wounds progress through the four stages of healing: hemostasis, inflammation, epithelial cell proliferation, and tissue remodeling (McCallon et al., 2014; Versey et al., 2021). In contrast, chronic wounds tend to arrest in the inflammation stage for at least four weeks to three months (Falcone et al., 2021). This results in an overactive neutrophil reactive oxygen species (ROS) response and extensive oxidative damage, loss of active fibroblasts, and a dysregulation of growth factors for the proliferation healing stage (Moser et al., 2021). Other virulence factors, such as the rhamnolipids produced by opportunistic bacterial pathogen *Pseudomonas aeruginosa*, contribute to tissue destruction by killing neutrophils and causing cellular necrosis (Moser et al., 2021).

The primary mechanism by which biofilms protect microbes against the host response is through immune evasion. At the start of their attempted invasion, microbes will first encounter innate immune defenses. The external surfaces of epithelial tissues are hostile environments for pathogen survival. In addition to acidity, desiccation, and competing with well-established commensal microbial populations, pathogens encounter ancient innate immune defenses, such as antimicrobial peptides (AMPs) (González et al., 2018; Coates et al., 2019). AMPs are amphipathic molecules which can target the negatively charged phosphate heads of the phospholipids in microbial cell membranes, leading to membrane disruption and cell lysis (Benfield and Henriques, 2020). However, eDNA and polysaccharides within a biofilm protect microbes from this innate immune defense by binding and sequestering charged molecules, including AMPs (Gunn et al., 2016). Once microbes breach the first line of defense, they will encounter additional innate immune components – the complement system, dendritic cells, macrophages, natural killer cells (NK), and polymorphonuclear cells (PMNs), including neutrophils (Gunn et al., 2016; González et al., 2018; Thomsen et al., 2022). The EPS provides a substantial barrier against immune attacks by binding both complement and immunoglobulins, therefore preventing opsonization and subsequent phagocytosis (Gunn et al., 2016). One of the simplest benefits that biofilms can provide microbial cells is an increase in size. The bulkiness of a biofilm can hamper or inhibit phagocytosis: aggregates above 5μm are too large to be engulfed by neutrophils (Thomsen et al., 2022). Furthermore, biofilms disguise microbes from immune detection. Immune cells, such as dendritic cells, macrophages, and monocytes, utilize pattern recognition receptors (PRRs) which recognize pathogen-associated molecular patterns (PAMPs) to distinguish self from invading microbes (González et al., 2018). In a biofilm, the majority of common PAMPs are concealed by EPS and other external biofilm structures (González et al., 2018). This allows biofilm microbes to slip by undetected and avoid activating key immune responses (González et al., 2018).

Biofilm-associated chronic infections in people with cystic fibrosis (CF) also cause overactive immune responses and damaging inflammation. In the airways of people with CF, the first responders are resident macrophages which detect invading microbes (Thomsen et al., 2022). If the resident macrophages encounter high concentrations of microbes (above 10^6^ CFU), as is the case in biofilms, PMNs will be recruited through PRRs (Thomsen et al., 2022). In CF chronic biofilm infections, highly mucoidal strains of *P. aeruginosa* produce high levels of the EPS component alginate which inhibits phagocytosis (Gunn et al., 2016). Unable to clear biofilms through phagocytosis, neutrophils will stagnate and surround the biofilm, employing other strategies such as ROS bursts and the release of proteolytic enzymes and NO that damage surrounding tissues (Jensen et al., 2010; Mauch et al., 2018; Thomsen et al., 2022). Lungs with large numbers of neutrophils in people with CF have been shown to have reduced lung functionality (Rada, 2017; Moser et al., 2021). An ineffective innate immune response will then trigger an adaptive immune response through the release of inflammatory cytokines and the activation of T helper cells by dendritic cells (González et al., 2018; Moser et al., 2021). A predominately Type 1 T helper (T_h_1) response produces IL-12, interferon gamma (INFγ), and tumor necrosis factor alpha (TNFα) to regulate phagocyte-dependent inflammation, macrophage activation, and cell-mediated immunity (González et al., 2018). In contrast, a prominent Type 2 T helper (T_h_2) response with high IL-4 levels influences immunoglobulin production through B cells (González et al., 2018). For people who have CF and chronic infections, poor prognosis is associated with skewed T_h_2 response with high antibody production; antibodies released from this response – primarily, excess IgG – form immune complexes that lead to ineffective and chronic inflammation that damages lung tissue (Jensen et al., 2010; González et al., 2018; Mauch et al., 2018; Moser et al., 2021).

#### Bulk mechanics of biofilms

**Figure 1:**
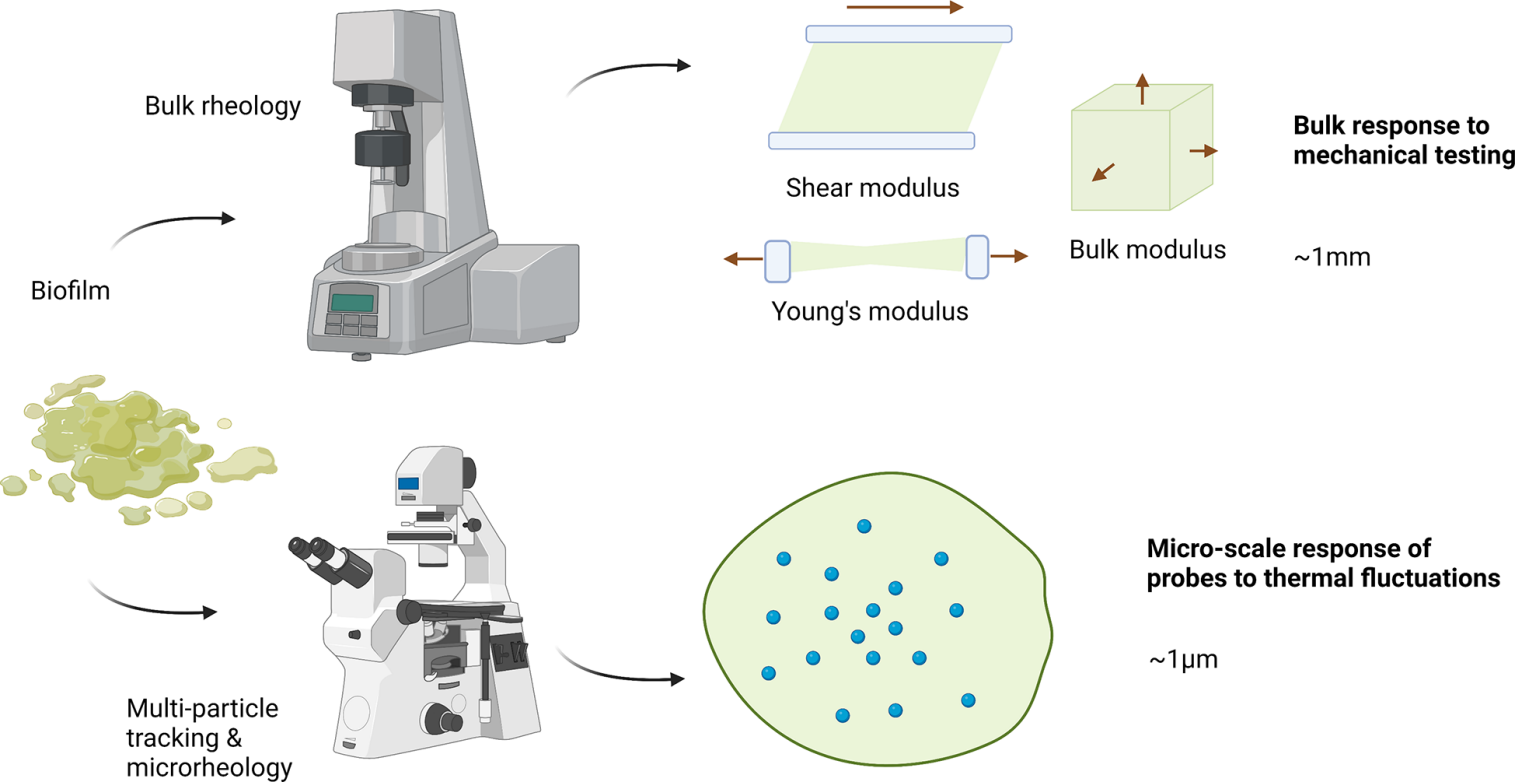
Bulk rheology and micro-rheology approaches for measuring biofilm mechanics. Typical bulk rheology grows a sample *ex situ*, which is then loaded into a holder and has a particular deformation applied. To measure shear moduli, the sample is typically placed in parallel plates ~ 1mm apart on a rotational rheometer. The plates apply a controlled stress or strain shear deformation, taking into account plate diameter and gap. For extension or compression measurements, typically a dynamic mechanical analyzer is used that has a range of sample holders which similar dimensions. In multi-particle tracking microrheology samples are grown in situ with particles within a sample holder. This system is then directly placed onto a microscope and particle motion is tracked. Typical particle sizes are ~ 1 micron. Created with BioRender.com.

Biofilms are heterogeneous materials with spatially and temporally variable structures. The production of multiple EPS components ensures that biofilms develop into complex viscoelastic materials. As such, mechanical analysis of biofilms by conventional means becomes difficult without disrupting the established structure (Stoodley et al., 2002). Moreover, as living systems, biofilms are highly variable between biological replicates, growth conditions, species, and strain. Understanding how biofilms respond to mechanical forces provides insight into their survival mechanisms and resistance to eradication. The mechanics of biofilms (among other materials) can be analyzed in terms of various intrinsic mechanical properties (**Figure 1**). At the macroscopic level, these include (this list is not exhaustive):

•Elastic modulus G: a material’s resistance to being deformed elastically, or a measure of stiffness. The elastic modulus can be defined in various ways according to the direction of the applied strain. For example:

⚬Young’s modulus: an object’s tendency to deform in either tension or compression

⚬Shear modulus: an object’s tendency to shear in response to anti-parallel forces (imposed in opposite directions on parallel planes)

⚬Bulk modulus: an object’s tendency to deform when forces are applied in all directions

•Yield point: the point of a stress-strain curve where plastic deformation begins

•Toughness: a measure of the amount of energy per unit volume that a material can absorb before permanent deformation or yielding

Biofilms that possess high elastic moduli are harder to deform, as they can withstand more mechanical stress while remaining in the reversible, linear-deformation regime. An increase in yield stress, which can be thought of as the force per area required to cause a biofilm to permanently deform or break, indicates an increase in the overall material strength.

Mechanical removal of biofilms has proven to be challenging. While *Streptococcus mutans* and *Staphylococcus epidermis* behave like viscous liquids when faced with high-speed microsprays (Gloag et al., 2020), *P. aeruginosa* forms stationary wrinkled structures, which indicate disruption of biofilm contact with substrate, but not the biofilm itself transitioning to fluid-like behavior. As such, mechanical disruption may ultimately lead to the spreading of infection.

We have shown that specific disruption of biofilms by polymer-degrading enzymes can alter the biofilm’s bulk mechanics (Kovach et al., 2020). *P aeruginosa* produces 3 dominant matrix polymers: alginate, Pel, and Psl, in addition to extracellular DNA. When grown from strains genetically modified to overproduce either alginate or Pel, the mechanics of biofilms are compromised when treated with alginate lyase (which breaks down glycosidic linkages) or DNAse (which cleaves phosphodiester bonds), respectively. Pel- and Psl- specific glycoside hydrolases have also been shown to cause dispersal of biofilms and increase antibiotic susceptibility (in both cases, for biofilms grown *in vivo*) (Fleming et al., 2017; Fleming and Rumbaugh, 2018; Fleming et al., 2020), albeit with minimal impact on mechanics for biofilms grown *in vitro* (Kovach et al., 2020).

Biofilm disruption has also recently been demonstrated by repurposing the human body’s natural defense mechanisms against *P. aeruginosa* biofilms (Louis et al., 2022). Human hormones have been shown to modulate bacterial response, and in a recent study, a human natriuretic peptide (a peptide hormone), hANP, demonstrated biofilm dispersal capabilities against *in vitro P. aeruginosa* biofilms by decreasing the proportion of β1-3 and β1-4 polysaccharides (Louis et al., 2022). Similar to other dispersal agents, hANP did not display bactericidal properties but could be utilized to augment antibiotic treatment (Louis et al., 2022).

Extracellular DNA is a known EPS component relied on by biofilms for structural integrity, though it may appear more prominently in different forms as the biofilm matures (Buzzo et al., 2021). B-DNA, the most common form of DNA found in nature and exhibiting the canonical right-handed double helix structure, is sensitive to nuclease degradation. However, B-DNA is capable of transforming into a nuclease-resistant form called Z-DNA, which has been found in more mature biofilms and may provide structural integrity without susceptibility to DNAse treatment.

Additionally, as a negatively charged polymer, DNA is capable of binding electrostatically with cations to form a cross-linked network which enhances the structural rigidity of the EPS system (Das et al., 2014; Hills et al., 2021; Cohen-Cymberknoh et al., 2022; Shokeen et al., 2022). Metal ions present in the airways of a person with CF, for example, may bind with both eDNA and alginate, which is also anionic. Calcium in particular has been observed as an exceptionally strong cross-linker, leading to increases in the Young’s modulus of an alginate-dominant biofilm. The presence of calcium has also been associated with an increase in the production of alginate, causing thicker, more mucous-like EPS. While sodium is the most common metal ion present in a CF-lung, it has been shown to be a relatively poor cross-linker compared to calcium. Sodium ions form weaker cross-links which are more readily broken, and much softer gel structures.

#### Phagocytic engulfment and the impact of physical properties of the target

Most cells are capable of endocytosing small molecules. The ability to engulf large particles is less common, occurring in specialized immune cells known as professional phagocytes. An actin-based process, phagocytosis is employed by immune cells such as neutrophils to clear particles greater than 0.5 microns in diameter. This process must be carefully regulated to avoid harmful host immune responses. The process of phagocytosis follows several phases: the detection of a target particle, the activation of the ingestion, the development of phagosome and its maturation to a phagolysosome, and the exposure of internalized targets to degrading enzymes (Uribe-Querol and Rosales, 2020). Receptor binding to antibody-coated objects is primarily responsible for initial recognition of phagocytic targets. Ligand binding causes receptor clustering and leads to signaling events such as tyrosine phosphorylation. Actin is subsequently recruited to begin the formation of the phagocytic cup (Beningo and Wang, 2002) and an attempt to phagocytose the target is initiated.

Phagocytic targets vary greatly in their physical properties, as does the behavior of phagocytes in response to these mechanical cues (Vorselen et al., 2020). When considering phagocytosis and biofilms, the mechanics of both the large-scale target and of the bacteria themselves are important. When faced with micron-sized polyacrylamide beads, neutrophils show a sixfold preference for engulfment of stiffer beads over their softer counterparts (Beningo and Wang, 2002). Indeed, while softer beads were more poorly ingested, they are found to adhere to the neutrophil’s surface, indicating that phagocytosis is inhibited by target mechanics after receptor binding. The mechanics of bacterial cells are regulated by a number of cellular and biochemical mechanisms, with the composition of the bacterial cell wall as a notable contributor. Gram-positive bacteria possess a thick layer of peptidoglycan and no outer membrane, while gram-negative bacteria have a thin peptidoglycan layer, a cytoplasmic membrane, and an outer membrane (Auer and Weibel, 2017). While gram-positive bacteria have been shown to be stiffer than their gram-negative counterparts (Tuson et al., 2012; Auer and Weibel, 2017), cell walls of gram-positive bacteria are found to be more viscous (Vadillo-Rodriguez et al., 2009). Preferential phagocytosis of gram-negative bacteria by amoeba (Rashidi and Ostrowski, 2019) may well be attributed to the viscoelastic properties of these cell walls.

#### Microrheology and spatial heterogeneities in the mechanics and microstructure of biofilms

The mechanical properties of biofilms are highly spatiotemporally heterogeneous and dynamic. Bacterial cells embedded in the matrix provide much of the structural material by secreting exopolysaccharides and eDNA, but the matrix also typically incorporates material from its environment. For example, *in vivo P. aeruginosa* biofilm matrices have been seen to extensively incorporate host environment collagen fibrils (Rahman et al., 2022a). These components generally combine in inhomogeneous distributions to create local regions of varying density, pore size, charge, and, consequently, viscoelasticity. Local differences can serve a practical purpose for the organism. Some regions of the biofilm matrix may be relatively tough to maintain attachment to a surface or protect the bacteria from mechanical disruption, while others might be softer, more porous channels through which water, nutrients, and antimicrobial substances selectively diffuse (Flemming et al., 2022).

With microrheological techniques (**Figure 1**), it’s possible to attain a fine-grain understanding of the structure of these local regions. Microrheology is also adaptable to hydrogel like materials which are softer (more viscous) or stiffer (more elastic). As such, these techniques can be applied to living biofilms, non-living biofilm models, and ionically-gelled biofilms, which possess unique structural characteristics of individual cells suspended in a gel of EPS polymers crosslinked with metal ions (for example, alginate with calcium) (Das et al., 2014; Hills et al., 2021; Jacobs et al., 2022). For softer materials, passive microrheology tracks the thermally driven motion of particles in the biofilm medium and relates it to properties such as the diffusion coefficient and relative viscoelasticity. Active microrheology, in which a magnet or optical tweezers are used to drive the motion of the particles, is suitable for stiffer materials (Gordon et al., 2017; Powell et al., 2021). Further, microrheology, compared to bulk rheology, can be performed *in situ* (Powell et al., 2021) and requires relatively minimal sample volume (Mason et al., 1997). Relative viscoelasticity is a parameter commonly connected to the structural characteristics of a biofilm, since more viscous character can be inferred to be a result of a less structured, more porous or flexible region. Relative viscoelasticity of biofilms is also correlated with biofilm survival and infectious morbidity (Rahman et al., 2021a). Hence, changes in microrheological mechanical characteristics such as relative viscoelasticity can be used as a metric to evaluate the effects of a variety of experimental manipulations on a biofilm’s structure.

The change in relative viscoelasticity of a biofilm before and after treatment with a candidate drug can provide a useful measure of the drug’s potential for use; increases in viscoelasticity may indicate a treatment’s success at disrupting the microstructures of the biofilm. Indeed, recent work has applied multiple particle tracking microrheology to demonstrate that increases in relative viscoelasticity values may be concomitant with a decrease in the heterogeneity of local viscoelasticity across a biofilm, and reflect successful disruption of the matrix superstructure (Powell et al., 2021).

Microrheology can also clarify the optimal targets for drug development. For biofilms grown *in vitro*, we have shown that strategic and efficient physical disruption of the biofilm matrix can be achieved by specifically targeting the components that make the most significant contributions to the biofilm’s mechanical properties (Kovach et al., 2020). Measurements of relative viscoelasticity can help implicate and evaluate specific matrix components in their structural roles and importance through exclusion and/or overproduction experiments.

### Recent work opening new questions

#### Mechanics of biofilms and phagocytic engulfment

It has been estimated that neutrophils and other phagocytes apply a stress of ~1kPa during phagocytosis (Evans et al., 1993; Herant et al., 2006; Jeong et al., 2007). 1kPa is within the range of elasticities we and others measure for *P. aeruginosa* biofilms grown *in vitro* (Kovach et al., 2017; Qi and Christopher, 2021). For *in vitro Staphylococcus aureus* and similar *Staphylococcus* spp biofilms, experimentally measured elasticity is ~10 Pa and viscosity is ~10 kPa (Shaw et al., 2004; Rupp et al., 2005). Comparing these moduli with the stresses exerted by phagocytosing neutrophils suggests that relatively-small changes in biofilm viscoelasticity are likely to impact the biofilms’ resistance to phagocytosis and to impact the timescale of phagocytosis, which could give more time for bacteria-produced toxins to kill neutrophils and other immune cells (Berube and Wardenburg, 2013; Alonzo and Torres, 2014; Cheung et al., 2014). Biofilm mechanics that delay or frustrate phagocytosis are also likely to lead to immune activity that is damaging to the host, as discussed above in *Host immune response to bacterial infection.*

On the bulk level, we have found that small rigid beads embedded in hydrogels are more readily engulfed when the gel’s elasticity is lower (Davis-Fields et al., 2019). As the target becomes large relative to the size of the neutrophil, the surface area of the neutrophil’s membrane is insufficient to engulf the target and close successfully close to form a phagosome (Vorselen et al., 2020). Certain mechanical properties of targets, such as toughness, may require extended timescales for phagocytic success (Bakhtiari et al., 2021), while others may not be overcome regardless of the timescale.

#### Microrheology of biofilms grown *in vivo*

Our earlier study on *P. aeruginosa* suggested that the biofilm’s growth environment could have a major influence on the success of enzymatic treatments targeted at bacterially secreted matrix polymers; at the time, we did not have an understanding of the causative linkage(s) between growth environment and biofilm matrix composition and mechanics (Kovach et al., 2020).

More recently, we found a considerable discrepancy between the relative viscoelasticity of biofilms grown *in vitro* and *in vivo*, pointing to a fundamental structural difference between the two as a consequence of differing growth environments. Further investigation suggested that the incorporation of collagen from the *in vivo* growth environment largely accounts for the differences, and can be the determining factor in *in vivo* biofilm viscoelasticity (Rahman et al., 2022a). These results together imply that previous *in vitro* experimental results may not be transferable to biological conditions, requiring future work in biofilm treatments to more extensively account for *in vivo* environmental influences. This is an important realization, since all studies of biofilm mechanics before our own recent work have been done for biofilms grown *in vitro*, and not in an actual infection (Shaw et al., 2004; Rupp et al., 2005; Kovach et al., 2017; Qi and Christopher, 2021).

### Illustrative example - Viscoelastic properties of *P. aeruginosa* biofilms

There are many different types of biofilm-forming pathogens, which can form biofilms in many different anatomical sites (as well as on medical instruments). The mechanics of these biofilms arises from the combination of the microbial species (one or more than one) involved as well as the specifics of the environment. What mechanical properties characterize biofilms of specific type and growth location is largely unknown; this is a wide-open field for study. There has been more progress made for *P. aeruginosa* biofilms than for biofilms of any other species, and therefore we present a summary of what is known about this pathogen’s biofilms as an illustration of the type of studies that could be done for other organisms and potential pitfalls to be avoided, if possible:

#### Distinct mechanical roles of different matrix components

EPS polysaccharides have multiple functionalities and redundancies, which makes isolating their impact on rheological properties of *P. aeruginosa* biofilms difficult to discern (Colvin et al., 2012). The use of mutant strains with EPS variations has enabled some conclusions about the effects of individual EPS components on viscoelasticity from bulk studies. For instance, Psl appears to increase elasticity, whereas Pel appears to increase relative viscosity, and alginate has been observed to increase yield strain (Kovach et al., 2017; Murgia et al., 2020).

#### Experimental concerns with bulk rheology: Sample preparation

One difficulty with biofilm rheology is that many sample preparation techniques homogenize a biofilm for bulk measurement. Therefore, the microstructure created by the EPS component, which determines the viscoelasticity, is modified by sample preparation. This makes results specific to sample preparation rather than the intrinsic microstructure of a biofilm grown *in-situ* or *in-vivo.* Ideally, in non-biological samples best practices include applying a pre-conditioning shear to samples before conducting actual tests. This pre-conditioning shear should essentially “erase” the deformation history arising from sample loading, so that all samples have similar microstructure before testing. However, such pre-conditioning is not typically recommended for biological samples - due to the complexity of biological samples, this procedure does not ensure similar microstructure across samples, and it still creates the same issue of modifying the native microstructure of the EPS before testing.

If using homogenized samples and loading into a rheometer, attempts should be made to deform the material as little as possible when loading it into the rheometer. Additionally, samples should be allowed some time to rest and reach a new equilibrium condition after loading. This can be tested by using small-amplitude oscillatory shear to measure the time scale it takes for the material to reach a steady-state response. Once this time is determined, all samples should be allowed to rest that long before studying. Obviously, this can create a problem in sustainability of the biofilm over the long term.

The best solution is to avoid these issues by growing biofilms in rheometers *in-situ*. This has been done for bulk rheology using specialized custom geometries (Pavlovsky et al., 2013). It can also be easily done using interfacial rheology when measuring pellicles (Rühs et al., 2013). However, both of these techniques are quite advanced and are not “standard” options with most rheometers; therefore, they require lab-specific custom equipment.

#### Experimental concerns with bulk rheology: Sample-to-sample variability

In addition, there is difficulty in interpreting and extrapolating the role of EPS components from bulk experiments because biofilms exhibit a large degree of sample-to-sample variability (Pavlovsky et al., 2013; Pavlovsky et al., 2015; Gloag et al., 2018; Horvat et al., 2019; Aravinda Narayanan and Ahmed, 2019). In our own work (Qi and Christopher, 2021), we have seen that variation in biofilms due to the sample-to-sample variation can be anywhere up to a factor of 10. Such sample-to-sample variation can come from sensitivity of the biofilms to environmental conditions, growth conditions, and/or intrinsic biological heterogeneity.

#### Advantages to microrheology

Particle tracking microrheology typically avoids both above problems. In comparison to typical bulk rheology, microrheology has a much more robust statistical significance. Typically, multiple particle tracking microrheology will examine similar numbers of biological replicates, but often will have more technical replicates and hundreds of particles tracked, creating data with robust statistical significance and overall larger sample sizes. Therefore, studies of the effects of specific EPS types on biofilm mechanics can be studied with greater confidence. Additionally, microrheology studies are typically done *in-situ*, meaning the microstructure of a biofilm is unchanged from the growth conditions.

Because of these advantages, there have been several studies that have examined how EPS polysaccharide components impact the viscoelasticity of *P. aeruginosa* biofilms using multiple particle tracking microrheology. These results have outlined in some detail the impact of each EPS component or EPS variation in general on viscoelasticity of *P. aeruginosa* biofilms. Below we outline those studies that specifically attacked this question.

#### Microrheology of lung homogenates

Muriga and coworkers (Murgia et al., 2020) used multi-particle tracking microrheology to study the viscoelasticity of lung homogenates from several mouse specimens. They found that despite these homogenates coming from the same bacterial source, there was a variation in observed viscoelasticity which was strongly correlated to severity of infection and bacterial biomarkers with increasing elasticity and stiffness correlating to higher CFU’s and severity. No specific examination of EPS components of the various homogenates was done, but it was theorized that the use of mucoid strains with increased alginate production and the general known changes of EPS composition in relation to host environment caused changed to the observed viscoelasticity. Although these results did not examine directly the role of individual EPS components on viscoelasticity, they posited that host changes to EPS composition affected the viscoelasticity.

#### Microrheology to examine the impacts of EPS types

The impacts of EPS components on viscoelasticity were more explicitly studied using multiple particle tracking microrheology by Chew and coworkers (Chew et al., 2014; Chew et al., 2015) (Chew et al., 2014; Chew et al., 2015). In this work, several strains of *P. aeruginosa* were studied that overexpressed alginate while producing no Pel, Psl, or both no Pel and Psl. Using confocal microscopy, the authors were able to examine how the various EPS affected both the overall viscoelasticity of the film. In this work, it was specifically found that Psl create stiffer more elastic biofilms due to increased crosslinking; alternatively, Pel created more viscous films that had “looser” microstructure. Note that these results are relatively like bulk studies as outlined earlier. However, as previously described, the larger sample sizes and ability to measure *in-situ* makes these findings more robust and potentially possible to extrapolate beyond this work.

In our own work studying this phenomenon (Rahman et al., 2021b; Rahman et al., 2022b), we looked at how the distribution of rheological properties, particularly relative elasticity and stiffness, changed with changes to EPS expression. In general it was noted that removing a single EPS polysaccharide through genetic knockouts impacted the stiffness/compliance and the relative elasticity of the biofilms different. Similar to Chew and coworkers, it was seen that Psl increased overall relative elasticity of the biofilm and decreased compliance, where as Pel seemed to create a more viscous overall film. The role of alginate in the biofilm mechanically was more difficult to pinpoint. Increased alginate production appeared to create more viscous films, whereas films made solely of alginate had larger yield strains. We should also note that most films showed similar mechanical evolution over time when a single component of EPS production was removed, which was like the results of Chew and coworkers. We also noted in general, films lacking polysaccharide components of the EPS were more homogenous than WT components. Finally, films with increased EPS production were general more elastic, stiffer, and more homogenous. The results in this work were quite consistent with the work of Chew and coworkers, however we were able to see in general that the individual EPS polysaccharides impacted relative viscoelasticity much more than overall biofilm stiffness, which is an important distinction. Furthermore, we noted that many of these changes were most impactful at early timescales vs. later times.

#### Beyond single-particle microrheology – multiple particle tracking

The above studies have used microrheology as a means of studying how EPS composition affects distribution and average response of rheological parameters. However, a more novel current approach uses multiple particle tracking microrheology and other similar techniques is to understand the spatial variation of biofilms rheological properties due to the distribution of EPS within the biofilm. This is relevant to EPS effects on viscoelasticity because EPS itself is heterogenous in its distribution throughout the biofilm. The exact composition/distribution of the EPS depends on the species/strain, gene expression, signaling, growth dynamics, chemotaxis, substrates composition, environmental factors, stochastic processes, and environmental stresses (Wimpenny et al., 2000). These factors create heterogeneity in the distribution of EPS components at length scales that can be interrogated by multiple particle tracking microrheology and several other techniques.

One of the earliest observations of such locally varying viscoelasticity was the work of Beyenal and coworkers (Beyenal et al., 1998), whom used a novel measurement technique to track particle diffusivity as a function of depth in the biofilm of *P. aeruginosa*. Although not converted to rheological values, particle diffusivity is specifically linked to biofilm viscoelasticity. A clear dependence was observed with diffusivity increasing as a function of distance from the substrate, indicating decreasing stiffness with depth. The results seemed to correlate to overall EPS distribution/density being larger near the substrate. These results indicated the power of multiple particle tracking microrheology to indicate how local composition manipulated local viscoelasticity.

Rogers and coworkers first showed that a similar technique could be used to understand the 2D spatial heterogeneity of *P. aeruginosa* biofilms (Rogers et al., 2008). In this work, multiple particle tracking microrheology was used to show statistically the great deal of variation in biofilms at a specific depth. The authors noted that they believed there was a link between spatial variation in EPS components and viscoelastic distribution. Chew and coworkers more explicitly explored this phenomenon (Chew et al., 2014; Chew et al., 2015). They were able to show that over time the more mature, core of a biofilm became less elastic and crosslinked, which was attributed to a reduction/degradation of Psl production. The younger outer extents of the biofilm remained more elastic and stiff because they still produced significant Psl. This study specifically shows the tremendous benefit of being able to map local EPS variation to local viscoelasticity using multiple-particle microrheology.

#### Combining particle tracking with other experimental techniques

Furthermore, these and similar techniques offer the ability to understand not just the role of individual EPS on the biofilm, but also the general microstructure. For instance, using nanoindentation (Baniasadi et al., 2014), variation of Young’s modulus across a 20x20 micron section of biofilm was seen to vary widely over 1x1 micron testing areas in a *P. aeruginosa* biofilm. Variation was able to be attributed to low EPS density/void spaces within the film. Using magnetic tweezers and active microrheology, Pavissich and coworkers (Pavissich et al., 2021) showed that local viscoelasticity heterogeneity was greater for *P. aeruginosa* grown at higher shear rates, which was an indication of changes to microstructure and EPS distributions.

#### Current standing and outlook for biofilm microrheology

In general, microrheology has enabled high statistically relevant measurements of EPS impacts on viscoelasticity and is increasingly being used to map the local viscoelasticity of biofilms, which can be used to understand local EPS distribution. However, currently the imaging of individual EPS components in real time hampers the ability to actual map EPS distributions to local viscoelasticity.

### Conclusion and future directions

**Figure 2:**
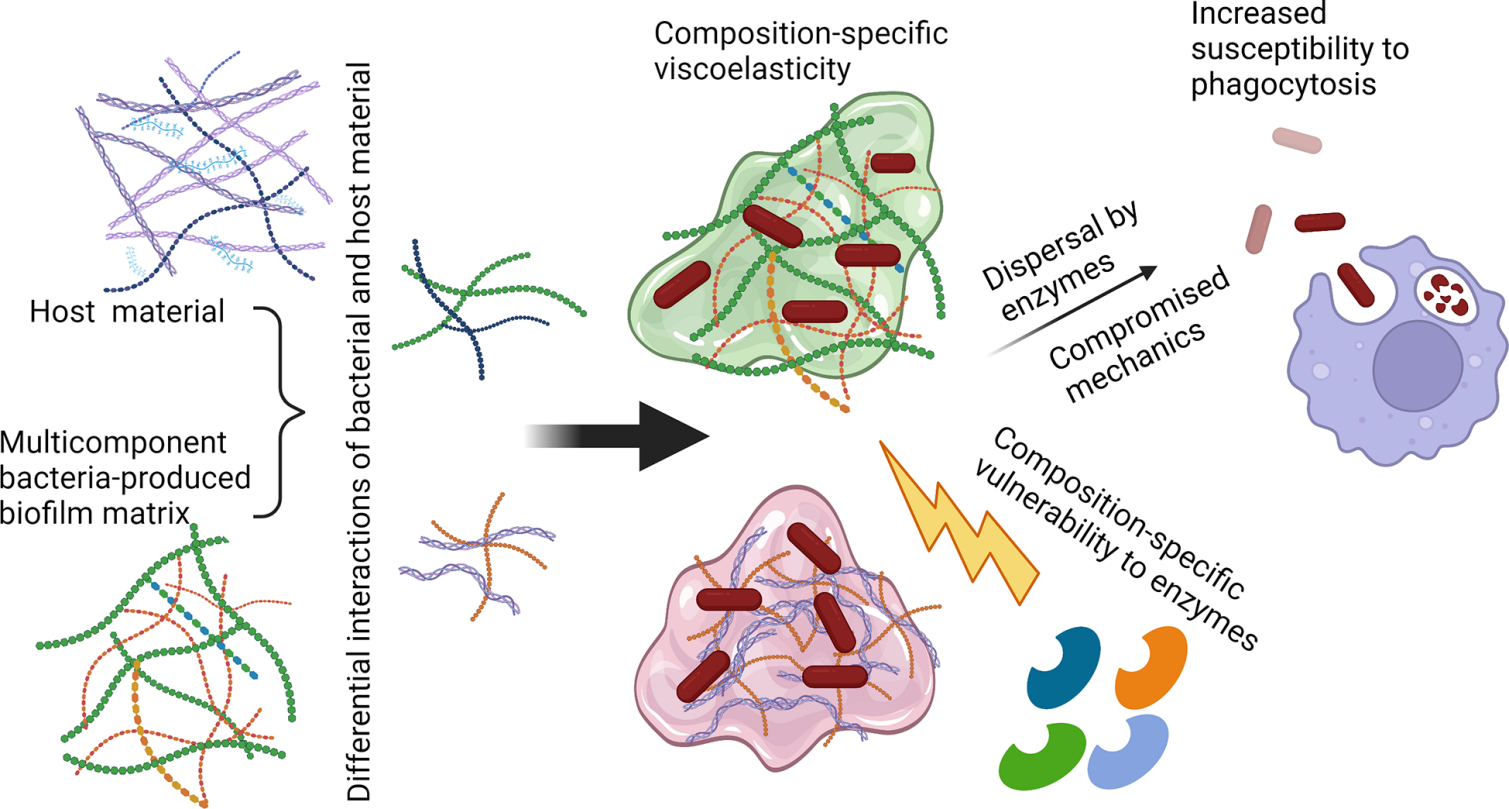
Possible route toward increasing biofilm susceptibility to phagocytic clearance by compromising matrix mechanics. Polysaccharide-degrading enzymes such as α-amylase (targeting α-1,4-glycosidic linkages) and cellulase (targeting β-1,4-glycosidic linkages), as well as nucleic acid-targeting enzymes such as DNase, could be utilized to disrupt the biofilm matrix and disperse bacterial cells. Release from the protective biofilm EPS could increase susceptibility to immune clearance through routes including neutrophil phagocytosis. Created with BioRender.com.

In short, the current state of the literature renders it plausible that the mechanical properties of biofilms may contribute to their evasion of the immune system, specifically to their resistance to phagocytic clearance. If so, this is likely to increase the harms done by immune infections themselves and by aspects of the inflammatory response that are damaging to the infected host. A promising pathway for elucidating this is to combine experimental measurements of phagocytic success (and other signatures of immune activities) with measurements of biofilm mechanics. Microrheology, for its ability to probe heterogeneous biofilms without perturbing their native structure, and for its ability to measure the mechanics of biofilms grown *in vivo*, is a promising tool for the latter. Revealing cases where compromising biofilm mechanics enhances phagocytic clearance (**Figure 2**) has the potential to open up a new class of therapeutic approaches that could be combined with treatments to promote bacterial dispersal and with conventional antimicrobials.

## Data availability statement

The original contributions presented in the study are included in the article/supplementary material. Further inquiries can be directed to the corresponding author.

## Author contributions

VG conceived the idea and scope for this Perspective. All authors contributed to the article and approved the submitted version.
